# Race, Ethnicity, and Mortality Following Major Osteoporotic Fracture: Results from the Women’s Health Initiative Study

**DOI:** 10.1007/s11606-025-09506-6

**Published:** 2025-04-24

**Authors:** Michaela Juels, Joseph C. Larson, Kristine E. Ensrud, Marcia L. Stefanick, Aladdin H. Shadyab, Lorena Garcia, Rami Nassir, Peter F. Schnatz, Rebecca Nelson, Carolyn J. Crandall

**Affiliations:** 1 David Geffen School of Medicine, University of California, Los Angeles, CA USA; 2 Fred Hutchinson Cancer Research Center, Seattle, WA USA; 3 Division of Epidemiology and Community Health and Department of Medicine, University of Minnesota, Minneapolis, MN USA; 4 Departments of Medicine (Stanford Prevention Research Center) and of Obstetrics & Gynecology, Stanford University, Stanford, CA USA; 5 Herbert Wertheim School of Public Health and Human Longevity Science and Division of Geriatrics, Gerontology, and Palliative Care, Department of Medicine, University of California San Diego, La Jolla, CA USA; 6 Department of Public Health Sciences, School of Medicine, University of California, Davis, CA USA; 7 Department of Pathology, School of Medicine, Umm Al-Qura University, Mecca, Saudi Arabia; 8 Departments of Obstetrics, Gynecology and Internal Medicine, Reading Hospital/Tower Health & Drexel University, Philadelphia, PA USA; 9 Department of Computational and Quantitative Medicine, Division of Biostatistics, City of Hope Comprehensive Cancer Center, Duarte, CA USA; 10 Division of General Internal Medicine and Health Services Research, Department of Medicine, David Geffen School of Medicine at University of California, Los Angeles, CA USA

**Keywords:** Osteoporosis, Women’s health, Social determinants of health, Health disparities, Major osteoporotic fracture

## Abstract

**Background:**

Major osteoporotic fracture (MOF) is associated with increased mortality; however, few studies in postmenopausal women have examined racial and ethnic differences in 1-year and 5-year mortality following MOF.

**Objective:**

To assess 1-year and 5-year mortality following MOF by race and ethnicity.

**Design:**

This prospective cohort study included postmenopausal women enrolled in the Women’s Health Initiative (WHI), a population-based, multisite US study. Participants were followed from September 1994 to February 2023. Data were analyzed between August 2023 and November 2023.

**Participants:**

Postmenopausal women aged 50 to 79 years old who experienced a MOF (*N* = 32,675 in 1 year and 29,506 in 5 years following MOF).

**Main Measures:**

Self-reported race and ethnicity. All-cause mortality was determined by death certificates, reports of surrogates, and the National Death Index Search.

**Key Results:**

The baseline mean age of participants was 77.0 [SD = 8.5] years with 31,223 [95.6%] White participants in the 1-year mortality analysis, and 76.3 [SD = 8.5] years with 28,212 [95.6%] White participants in the 5-year mortality analysis. In fully adjusted models, compared to White women, Black women had a higher risk of mortality (adjusted odds ratio (aOR) = 1.42, 95% CI [1.06, 1.90], while Asian women had a lower risk of mortality (aOR = 0.48 95% CI [0.27, 0.88]), within 1 year following MOF. Compared to White women, the mortality risk within 5 years after MOF was significantly higher among American Indian/Alaska Native (aOR = 3.30, 95% CI [1.65, 6.60]) and lower among Asian (aOR = 0.58, 95% CI [0.42,0.80]) women. While there were no mortality differences by ethnicity 1 year following MOF, Hispanic/Latina women were less likely to die 5 years following MOF (aOR = 0.74, [95% CI 0.57–0.96]) compared to Non-Hispanic/Latina women.

**Conclusions:**

In this large prospective study, mortality following MOF differed by race. Future research is needed to delineate the mechanism behind these associations.

**Supplementary Information:**

The online version contains supplementary material available at 10.1007/s11606-025-09506-6.

## INTRODUCTION

Osteoporosis is a disease characterized by low bone mass, leading to enhanced bone fragility and increased fracture risk.^1^ Following an osteoporotic fracture, there is a higher risk of subsequent fracture, morbidity, and mortality.^2–6^ Racial/ethnic disparities may exist in the evaluation and management of osteoporosis. A retrospective cohort study of 399,000 postmenopausal osteoporotic women living in the USA found that, compared to White women, Black women had a significantly higher risk of age-adjusted mortality 1 year after femur, hip, humerus, and radius/ulna fractures. The reasons for this discrepancy are unclear.^7^ To our knowledge, previous studies have not described the associations between other races and ethnicities following major osteoporotic fractures (MOF; hip, clinical spine, upper arm/shoulder, lower arm/wrist fracture).

To address this knowledge gap, we compared 1-year and 5-year mortality following MOF by race and ethnicity. We hypothesized that the risk of mortality following MOF would be greater in non-White women compared to White women and in Hispanic/Latina women compared to non-Hispanic women.

## METHODS

### Study Participants

Between 1993 and 1998, the Women’s Health Initiative (WHI) enrolled 161,808 postmenopausal women aged 50 to 79 years old at 40 US clinical centers.^8^ Exclusion criteria included predicted survival time of less than 3 years or conditions or characteristics interfering with study participation (alcoholism, mental illness, dementia). The WHI Observational Study (WHI-OS) examined the predictors and causes of morbidity and mortality in postmenopausal women. The WHI Clinical Trials (WHI-CT) tested a low-fat diet, menopausal hormone therapy, and calcium and vitamin D supplementation. The main study was carried out between 1993 and 2005.^8^ Following the main WHI-CT and WHI-OS (1993–2005), all active study participants were invited to continue participation for 5 years at their respective clinical centers (Extension Study 1, 2005–2010). From 2010 to present, those still actively participating were invited to continue ongoing follow-up in the WHI Extension Study. This study includes data from September 1994 to February 2023. Institutional review board approval was obtained at each center. All participants provided written informed consent.

### Assessment of Race/Ethnicity

Race and ethnicity were self-reported through a baseline self-assessment questionnaire. Participants from 1993 to 1998 were asked to “describe your race or ethnic group” with the following choices: (1) American Indian or Alaska Native; (2) Asian or Pacific Islander; (3) Black or African American (not of Hispanic origin); (4) Hispanic/Latino; (5) White (not of Hispanic origin); and (6) Other (Specify). From 2003 onwards, participants were asked “Are you Spanish/Hispanic/Latino?” Next, participants were asked “What is your race? Mark one or more races to indicate what you consider yourself to be” with the following response choices: (1) White; (2) Black, African-American; (3) American Indian or Alaska Native; (4) Asian Indian; (5) Chinese; (6) Filipino; (7) Japanese; (8) Korean; (9) Vietnamese; (10) Other Asian; (11) Native Hawaiian; (12) Guamanian or Chamorro; (13) Samoan; (14) Other Pacific Islander; (15) Some other race. This analysis followed the guidelines in the WHI Race and Ethnicity Language and Data Interpretation Guide in which Ethnicity is defined as (1) “Hispanic or Latino” or “Not Hispanic or Latino,” and race is defined as (1) African American or Black; (2) American Indian or Alaskan Native; (3) Asian; (4) Native Hawaiian and Pacific Islander; and (5) White.^9,10^

### Assessment of MOF

Participants were asked if they had been told by a healthcare provider that they had a new broken, fractured, or crushed hip, upper leg bone, or bone other than the hip or leg. Participants who marked yes were instructed to identify the location of the fractured bone (hip, lower arm/wrist, spine, or upper arm/shoulder fracture.)

MOF was defined as a hip, lower arm/wrist, clinical spine, or upper arm/shoulder fracture. MOF of the lower arm/wrist, spine, or upper arm/shoulder fracture was self-reported only. Hip fractures were initially self-reported (semiannually in WHI-CT, annually in WHI-OS) and subsequently confirmed by a physician adjudicator using medical records from baseline until 2010. During the WHI extension study 2 (2010 to present), the WHI population was divided into the Medical Records Cohort (MRC) and the Self-Report Cohort (SRC). Only the MRC had hip fractures confirmed by a physician adjudicator using medical record review, while for the SRC hip fractures were self-reported.

**Figure 1:**
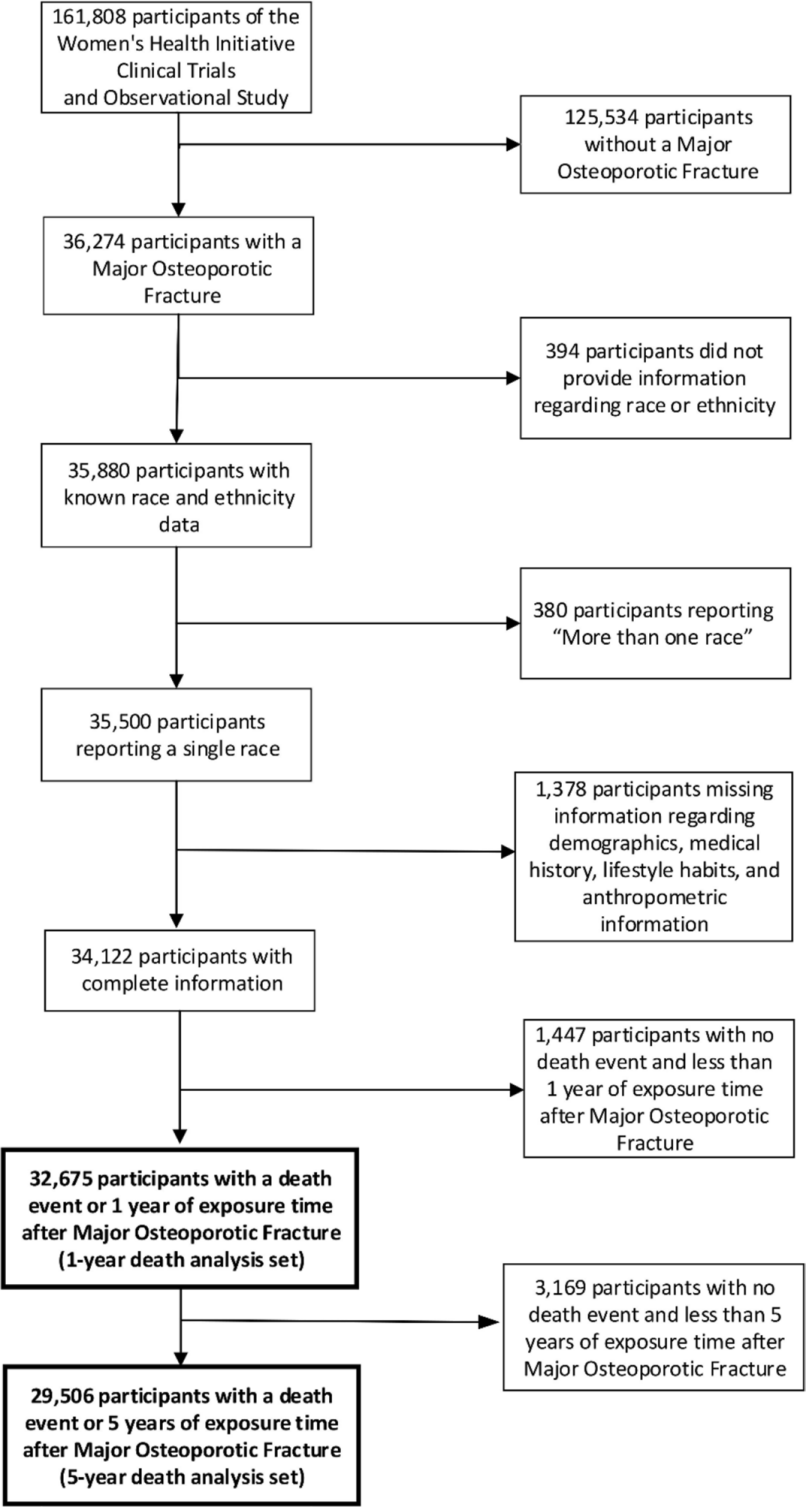
STROBE diagram.

Of 161,808 participants in the WHI-OS and WHI-CTs, we excluded data from participants who did not have an MOF event (*n* = 125,534), participants who did not provide information regarding race or ethnicity (*n* = 394), participants who reported more than one race (*n* = 380), and participants for whom we lacked complete information regarding covariates (*n* = 1378) (Fig. 1). For our 1-year mortality analysis, we excluded participants with no death event and less than 1 year of exposure time after MOF event (*n* = 1447). For our 5-year mortality analysis, we excluded participants with no death event and less than 5 years of exposure time after MOF event (*n* = 3169). To avoid bias in excluding participants who had death dates on the same date as their MOF event, those participants had their death date set to a half day after their MOF date and were included in all analyses. Therefore, the analytic sample for the 1-year mortality analysis included 32,675 participants and the 5-year mortality analysis included 29,506 participants.

### Outcomes

Mortality was determined by death certificates, reports of surrogates, and the National Death Index Search (NDIS). For all participants in WHI, Extension 1, and MRC participants in Extension 2, deaths were adjudicated by a physician using death certificates and medical record review. For SRC participants in Extension 2, information on death was enhanced by annual NDIS information.

### Other Covariates

Information regarding age, race/ethnicity, educational level, tobacco smoking, alcohol intake, and medication use were obtained using a baseline self-assessment questionnaire. The RAND 36-item health survey physical functioning score and the RAND 36-item survey well-being score were calculated (range from 0 to 100, with higher scores indicating more favorable well-being state).^11^ On self-assessment questionnaires, participants were asked about current smoking and current alcohol intake. These covariates were assessed as close to MOF event as possible (without being after the MOF). Timepoints of covariates assessment are displayed in Table 1. Height and weight were measured at baseline. Body mass index (BMI) was calculated as body weight in kilograms (kg) divided by the square of height in meters (m).

**Table 1:** Characteristics of Participants Overall and by 1-Year Mortality

Demographic	All participants (*n* = 32,675)		1-year mortality				
			No (*n* = 30,970)		Yes (*n* = 1705)		
	*n*	%	*n*	%	*n*	%	*p*-value
Age, years, mean (SD)	77.0	(8.5)	76.6	(8.4)	83.5	(8.1)	< 0.001
< 70	6349	19.4	6249	20.2	100	5.9	
70–80	14,598	44.7	14,138	45.7	460	27.0	
> 80	11,728	35.9	10,583	34.2	1145	67.2	
Race							0.08
American Indian/Alaska Native	53	0.2	49	0.2	4	0.2	
Asian	446	1.4	434	1.4	12	0.7	
Native Hawaiian/Pacific Islander	11	0.0	11	0.0	0	0.0	
Black/African American	942	2.9	885	2.9	57	3.3	
White	31,223	95.6	29,591	95.5	1632	95.7	
Ethnicity							0.10
Not Hispanic/Latina	32,015	98.0	30,335	97.9	1680	98.5	
Hispanic/Latina	660	2.0	635	2.1	25	1.5	
WHI study component							0.40
Clinical trial	14,198	43.5	13,474	43.5	724	42.5	
Observational study	18,477	56.5	17,496	56.5	981	57.5	
MOF fracture site^a^							
Hip	6248	19.1	5568	18.0	680	39.9	< 0.001
Spine	8612	26.4	8103	26.2	509	29.9	< 0.001
Upper arm/shoulder	6567	20.1	6295	20.3	272	16.0	< 0.001
Lower arm/wrist	11,928	36.5	11,630	37.6	298	17.5	< 0.001
Medical history							
Cancer	7149	21.9	6470	20.9	679	39.8	< 0.001
CVD	4334	13.3	3854	12.4	480	28.2	< 0.001
MI	1752	5.4	1576	5.1	176	10.3	< 0.001
CABG	844	2.6	750	2.4	94	5.5	< 0.001
PTCA	1621	5.0	1472	4.8	149	8.7	< 0.001
Stroke	1700	5.2	1466	4.7	234	13.7	< 0.001
COPD	4374	13.4	4047	13.1	327	19.2	< 0.001
Treated diabetes mellitus	4542	13.9	4170	13.5	372	21.8	< 0.001
Education							0.03
≤ High school/GED	6424	19.7	6047	19.5	377	22.1	
School after high school	12,205	37.4	11,587	37.4	618	36.2	
College degree or higher	14,046	43.0	13,336	43.1	710	41.6	
Region							0.04
Northeast	8424	25.8	8004	25.8	420	24.6	
South	7531	23.0	7152	23.1	379	22.2	
Midwest	7423	22.7	7055	22.8	368	21.6	
West	9297	28.5	8759	28.3	538	31.6	
BMI, kg/m^2^, mean (SD)	27.6	(5.7)	27.6	(5.7)	27.3	(5.7)	0.06
Physical function^b^ (0–100), mean (SD)	74.6	(23.4)	75.1	(23.1)	65.3	(26.3)	< 0.001
Emotional well-being^a^ (0–100), mean (SD)	78.8	(14.6)	78.9	(14.6)	77.9	(15.2)	0.005
Smoking							0.02
Never	16654	51.0	15833	51.1	821	48.2	
Past	14756	45.2	13953	45.1	803	47.1	
Current	1265	3.9	1184	3.8	81	4.8	
Alcohol use							< 0.001
Never	3078	9.4	2904	9.4	174	10.2	
Past	6382	19.5	5988	19.3	394	23.1	
Current	23,215	71.0	22,078	71.3	1137	66.7	
Medication use							
Osteoporosis medications	9204	28.2	8744	28.2	460	27.0	0.26
Bisphosphonates	7622	23.3	7244	23.4	378	22.2	0.25
Calcitonin	963	2.9	903	2.9	60	3.5	0.15
Parathyroid hormones	64	0.2	63	0.2	1	0.1	0.19
SERMs	1420	4.3	1352	4.4	68	4.0	0.46
Rank ligand inhibitors	4	0.0	3	0.0	1	0.1	0.08

*Abbreviations*: *MOF*, major osteoporotic fracture; *CVD*, cardiovascular disease; *MI*, myocardial infarction; *CABG*, coronary artery bypass graft; *PTCA*, percutaneous transluminal coronary angioplasty; *COPD*, chronic obstructive pulmonary disease; *GED*, general educational development; *BMI*, body mass index; *SERMs*, selective estrogen receptor modulator; *RAND SF- 36*, 36-Item Short Form Health Survey; *WHI*, Women’s Health Initiative

^a^660 participants had fractures at multiple sites

^b^Assessed using the RAND SF- 36 survey

### Statistical Analysis

We examined key characteristics of participants at the time of their MOF event overall and by mortality status, using means and standard deviations for continuous variables, and frequencies and percentages for categorical variables. *p*-values comparing differences by mortality status were calculated using *t*-tests for continuous variables and Chi-square tests for categorical variables. Additionally, we examined participant characteristics by race and ethnicity.

Logistic regression modeling was used to evaluate the relationships of self-reported race/ethnicity with 1-year and 5-year mortality after MOF. To avoid bias in excluding participants who had death dates on the same date as their MOF event, those participants had their death date set to a half day after their MOF date and were included in all analyses.

Fully adjusted models accounted for age, WHI study component (observational study or clinical trial), educational attainment, geographic region, history of cancer (other than non-melanoma skin cancer), history of cardiovascular disease (non-fatal myocardial infarction, fatal myocardial infarction, fatal stroke, non-fatal stroke, percutaneous transluminal coronary angioplasty), history of chronic obstructive pulmonary disease, history of treated diabetes mellitus, current smoking, current alcohol intake, BMI, RAND SF- 36 physical function score, RAND SF- 36 emotional well-being score, and ever-use of prescription osteoporosis medication (bisphosphonates, calcitonin, parathyroid hormone, selective estrogen receptor modulators, receptor activator of nuclear factor kappa-Β (RANK) ligand inhibitors). All models evaluating mortality by race were adjusted for ethnicity; models evaluating mortality by ethnicity were adjusted for race.

Results are presented using odds ratios (OR), corresponding 95% confidence intervals (CIs), and *p*-values testing for differences across all groups. Two-sided *p*-values less than 0.05 were considered statistically significant.

Analyses were carried out using SAS for Windows v.9.4 (SAS Institute).

## RESULTS

### Participant Characteristics

The mean (SD) participant age at the time of the MOF event of the 32,675 participants in the 1-year mortality analytic cohort was 77.0 (8.5) years (Table 1). Fifty-three participants (0.2%) self-identified as American Indian/Alaska Native; 446 participants (1.4%) self-identified as Asian; 942 participants (2.9%) self-identified as Black/African American; 11 participants (0.0%) self-identified as Native Hawaiian/Pacific Islander; 31,223 participants (95.6%) self-identified as White. Six-hundred sixty (2.0%) of participants self-identified as Hispanic/Latina and 3205 (98.0%) self-identified as not Hispanic/Latina.

Compared to women who survived 1 year after MOF event, women who died were significantly older (83.5 (SD = 8.1) vs. 76.6 (SD = 8.4) years, *p* < 0.001). Compared to participants who did not die within 1 year after MOF, the participants who died had significantly higher proportion of MOF fractures occurring at the hip (39.9% vs. 18.0%, *p* < 0.001) and a lower proportion of MOF fractures occurring at the lower arm/wrist (17.5% vs. 37.6%, *p* < 0.001) (Table 1). Generally, participants with (vs. without) comorbid medical conditions were more likely to experience a death event 1 year following MOF. Also, participants who died within 1 year after MOF compared to participants who did not die had significantly lower physical function scores an average of 7 years prior to MOF (65.3 vs. 75.1, *p* < 0.001). Mean BMI was similar among participants who died and those who did not die within 1 year after MOF. Compared to participants who died within 1 year after MOF, participants who did not die had a significantly higher prevalence of current alcohol use (71.3% vs. 66.7%, *p* < 0.001). These associations were comparable in the 5-year analytic cohort (eTable 1 in Supplement 1).

Compared to Hispanic/Latina participants at the time of the MOF event, non-Hispanic/Latina participants had a higher proportion of MOFs at the hip (19.3% vs 9.7%), but a lower proportion of MOFs at the upper arm/shoulder MOFs (19.9% vs 27.4%) (eTable 2 in Supplement 1). At the time of the MOF event, White compared to non-White participants had a higher proportion of MOFs at the hip (19.4%) (eTable 3 in Supplement 1).

### MOF Fracture Sites by Race and Ethnicity

MOF fracture site distribution differed by race (eTable 3 in Supplemental 1). Lower arm/wrist fractures were the most common fracture type across all racial groups, occurring in 5 (45.5%) Native Hawaiian/Pacific Islander women, 420 (44.6%) Black/African American women, 176 (39.5%) Asian women, 29 (54.7%) American Indian/Alaska Native women, and 11,298 (36.2%) White women. Upper arm/shoulder fractures were more frequent in 4 (36.4%) Native Hawaiian/Pacific Islander women and 253 (26.9%) Black/African American women compared to 6226 (19.9%) White women. Hip fractures were most prevalent in 6047 (19.4%) White women and least common in 1 (9.1%) Native Hawaiian/Pacific Islander woman. Spine fractures were most common in Asian women (136 [30.5%]) and White women (8298 [26.6%]), with lower rates in Black/African American women (165 [17.5%]), American Indian/Alaska Native women (12 [22.6%]), and Native Hawaiian/Pacific Islander women (1 [9.1%]).

Fracture patterns also varied by ethnicity (eTable 2 in Supplemental 1). Compared to 32,015 non-Hispanic/Latina women, the 660 Hispanic/Latina women had fewer hip fractures (64 [9.7%] vs. 6184 [19.3%]), but more upper arm/shoulder (181 [27.4%] vs. 6386 [19.9%]) and lower arm/wrist fractures (272 [41.2%] vs. 11,656 [36.4%]).

### Associations of Race and Ethnicity With 1-Year Mortality Following MOF

In fully adjusted models, the risk of mortality within 1 year after MOF was significantly higher in Black versus White women (OR = 1.42, 95% CI [1.06, 1.90]) (Table 2). Asian compared to White women had lower risk of mortality (OR = 0.48, 95% CI [0.27, 0.88]) within 1 year following MOF. No significant association was found between ethnicity (Hispanic/Latina or Not Hispanic/Latina) and 1-year mortality after MOF (eTable 4 in Supplement 1).

**Table 2:** 1-Year Mortality Following Major Osteoporotic Fracture by Race

	Race					
	American Indian/Alaska Native	Asian	Native Hawaiian/Pacific Islander	Black/African American	White	
*n*	53	446	11	942	31223	
Events	4	12	0	57	1632	
%	7.5	2.7	0.0	6.1	5.2	
Model^a^	OR (95% CI)	OR (95% CI)	OR (95% CI)	OR (95% CI)	OR (95% CI)	*p*-value
Model 1	2.17 (0.76, 6.22)	0.46 (0.26, 0.83)	-	1.64 (1.24, 2.19)	1.00 (ref)	< 0.001
Model 2	1.84 (0.63, 5.41)	0.49 (0.27, 0.88)	-	1.56 (1.17, 2.09)	1.00 (ref)	0.003
Model 3	1.79 (0.62, 5.21)	0.47 (0.26, 0.85)	-	1.45 (1.08, 1.94)	1.00 (ref)	0.009
Model 4	1.81 (0.62, 5.27)	0.48 (0.27, 0.88)	-	1.42 (1.06, 1.90)	1.00 (ref)	0.02

*Abbreviations*: *OR*, odds ratio; *CI*, confidence interval; *WHI*, Women’s Health Initiative

^a^All logistic regression models are adjusted for ethnicity

Model 1: Adjusted for fracture site, WHI study component (observational study or clinical trial), age, education, and region

Model 2: Model 1 + history of cancer, history of cardiovascular disease, history of chronic obstructive pulmonary disease, history of treated diabetes mellitus

Model 3: Model 2 + current smoking, current alcohol use, body mass index, physical function score, emotional well-being score

Model 4: Model 3 + ever-used osteoporosis medication

### Associations of Race and Ethnicity with 5-Year Mortality Following MOF

In fully adjusted models, the risk of mortality within 5 years after MOF was significantly higher in American Indian/Alaska Native than White women (OR = 3.30, 95%CI [1.65, 6.60], *p* = < 0.001) (Table 3). After adjustment for fracture site, WHI study component (observational study, clinical trial), age, education, and geographic region, compared to White women, Black versus White women had a higher risk of dying within 5 years after MOF (OR = 1.23, 95%CI [1.01, 1.50], *p* = < 0.001); however, this association was substantially attenuated and not statistically significant in fully adjusted models (OR = 0.98, 95% CI [0.80, 1.20]. In fully adjusted models, Asian compared to White women had a lower risk of mortality (OR = 0.58, 95% CI [0.42, 0.80]) within 5 years following MOF. There was a significant association between ethnicity and 5-year mortality, with Hispanic/Latina versus non-Hispanic/Latina women being less likely to die within 5 years after MOF (adjusted OR = 0.74, [95%CI 0.57–0.96], *p* = 0.03) (Table 4).

**Table 3:** 5-Year Mortality After Major Osteoporotic Fracture (MOF) by Race

	Race					
	American Indian/Alaska Native	Asian	Native Hawaiian/Pacific Islander	Black/African American	White	
*n*	45	388	9	852	28212	
Events	17	56	1	164	6290	
%	37.8	14.4	11.1	19.2	22.3	
Model^a^	OR (95% CI)	OR (95% CI)	OR (95% CI)	OR (95% CI)	OR (95% CI)	*p*-value
Model 1	4.27 (2.18, 8.36)	0.53 (0.38, 0.72)	0.94 (0.10, 9.16)	1.23 (1.01, 1.50)	1.00 (ref)	< 0.001
Model 2	3.77 (1.89, 7.50)	0.55 (0.40, 0.75)	1.15 (0.12, 10.87)	1.11 (0.91, 1.36)	1.00 (ref)	< 0.001
Model 3	3.25 (1.62, 6.49)	0.56 (0.40, 0.78)	1.35 (0.15, 12.40)	1.00 (0.81, 1.23)	1.00 (ref)	< 0.001
Model 4	3.30 (1.65, 6.60)	0.58 (0.42, 0.80)	1.36 (0.15, 12.43)	0.98 (0.80, 1.20)	1.00 (ref)	< 0.001

*Abbreviations*: *OR*, odds ratio; *CI*, confidence interval; *WHI*, Women’s Health Initiative

^a^All logistic regression models are adjusted for race

Model 1: Adjusted for fracture site, WHI study component (observational study or clinical trial), age, education, and region

Model 2: Model 1 + history of cancer, history of cardiovascular disease, history of chronic obstructive pulmonary disease, history of treated diabetes mellitus

Model 3: Model 2 + current smoking, current alcohol use, body mass index, physical function score, emotional well-being score

Model 4: Model 3 + ever-used osteoporosis medication

**Table 4:** 5-Year Mortality After Major Osteoporotic Fracture (MOF) by Ethnicity

	Ethnicity		
	Not Hispanic/Latina	Hispanic/Latina	
*N*	28,926	580	
Events	6437	91	
%	22.3	15.7	
Model^a^	OR (95% CI)	OR (95% CI)	*p*-value
Model 1	1.00 (ref)	0.75 (0.58, 0.97)	0.03
Model 2	1.00 (ref)	0.75 (0.58, 0.97)	0.03
Model 3	1.00 (ref)	0.75 (0.58, 0.97)	0.03
Model 4	1.00 (ref)	0.74 (0.57, 0.96)	0.03

*Abbreviations*: *OR*, odds ratio; *CI*, confidence interval; *WHI*, Women’s Health Initiative

^a^All logistic regression models are adjusted for race

Model 1: Adjusted for fracture site, WHI study component (observational study or clinical trial), age, education, and region

Model 2: Model 1 + history of cancer, history of cardiovascular disease, history of chronic obstructive pulmonary disease, history of treated diabetes mellitus

Model 3: Model 2 + current smoking, current alcohol use, body mass index, physical function score, emotional well-being score

Model 4: Model 3 + ever-used osteoporosis medication

### Mortality After MOF According to MOF Fracture Site

In fully adjusted models, compared to those who experienced lower arm/wrist MOF, participants who experienced hip MOF were 2.8-fold more likely to die within 1 year of MOF (95%CI [2.40, 3.25] (eTable 5 in Supplement 1) and 2.2-fold more likely to die within 5 years after MOF (95%CI [2.05, 2.45]) (eTable 6 in Supplement 1). The sample size was insufficient across racial groups to evaluate how race and ethnicity impacted the type of MOF.

## DISCUSSION

This prospective study compared the risk of mortality 1 year and 5 years following MOF in different racial and ethnic groups of postmenopausal women. We found that mortality 1 year following MOF was substantially higher among Black women and lower among Asian women compared to White women. Additionally, 5-year mortality following MOF was substantially higher among American Indian/Alaska Native women (and lower among Asian women) compared to White women and lower among Hispanic/Latina women compared to non-Hispanic/Latina women.

Disparities in clinical outcomes following MOF are the focus of increasing attention. We cannot compare the current results regarding mortality following MOF by the other races or by ethnicity with those of previously published studies because, to our knowledge, studies that examined mortality following MOF did not evaluate this relationship according to race and ethnicity.^6,12,13^ However, our findings of increased mortality 1-year following MOF among Black women are consistent with findings of a previous observational cohort study of postmenopausal osteoporotic women that reported that Black compared with White women had a significantly higher risk of age-adjusted mortality 1 year after femur, hip, humerus, and radius/ulna fractures.^7^ The previous study did not examine 5-year mortality or other categories of race such as American Indian or Alaskan Native, Asian, or Native Hawaiian and Pacific Islander, or ethnicity.

To our knowledge, there are no studies comparing mortality following MOF in Asian and American Indian/Alaska Native women compared to White women. The mechanisms explaining differences in post-fracture mortality between Black and American Indian/Alaska Native women compared to White women are unclear. For example, in our study, White women had higher rates of MOF at the hip (19.4%) compared to Asian, American Indian/Alaskan Native, and Black women. In general, hip fractures are associated with higher mortality compared to other types of MOF, such as upper arm/shoulder or vertebral fractures;^13^ therefore, our current findings of higher mortality following MOF among Black and American Indian/Alaska Native women compared to White women are not explained by differences in MOF fracture sites between races. One possible explanation is the lower use of osteoporosis medications among Black (15.2%) and American Indian/Alaska Native women (9.1%) compared to White women (28.4%) (eTable 3 in Supplement 1). Untreated osteoporosis following MOF can increase the risk of subsequent fracture and mortality following initial fracture.^14,15^ Future research should investigate whether differences in osteoporotic treatment contribute to racial disparities in post-fracture mortality. Disparities in osteoporosis screening and limited access to rehabilitation services following hip fractures may contribute to the increased MOF mortality risk observed in Black and American Indian/Alaska Native women.^16–18^ However, because these racial groups had a much lower risk of hip fracture compared to White women, access to rehabilitation services likely does not explain our findings. Also, we must acknowledge that, compared to White women, Asian women had lower mortality at both 1 and 5 years after MOF. Addressing these differences requires structural policy changes, improved access to osteoporosis care, and future research to delineate underlying mechanisms.

The differences in post-fracture mortality between Asian and White women may be explained in part by the variation in MOF skeletal sites between these two racial groups. White women experienced a higher incidence of hip fractures (19.4%) compared to Asian women (14.8%). Indeed, hip fractures are associated with higher mortality compared to other types of MOF.^13^ Additionally, Asian women compared to White women had a higher incidence of MOF at the spine (30.5% vs. 26.6%) and arm/wrist (39.5% vs. 36.2%). While women with diagnosed vertebral fractures generally have higher mortality than those without such fractures^19^ mortality after spine fracture is lower than that after hip fracture.^20^ These findings suggest that the lower 1-year and 5-year mortality rates following MOF among Asian women, compared to White women, may be partially attributed to differences in distribution of MOF fracture sites.

In this study, 5-year mortality following MOF was lower among Hispanic/Latina women compared to non-Hispanic/Latina women. This finding may be explained by differences in MOF fracture sites by ethnicity. For example, compared to Hispanic/Latina women, non-Hispanic/Latina women had a higher percentage of MOF fractures at the hip, which are associated with higher mortality than other types of MOF.

Our findings are a first step toward understanding and reducing disparities in clinical outcomes following MOF. Our results have implications for clinical care following MOF, highlighting that greater attention should be given to potential race- and ethnicity-specific differences in mortality following MOF. Potential differences in fracture management after MOF across racial and ethnic groups are an important target of future research to delineate the mechanisms for these mortality differences and inform interventions to improve outcomes following MOF across racial and ethnic groups.

Strengths of our study include a large racially and ethnically diverse group of postmenopausal women, prospective follow-up of participants, and detailed information regarding osteoporosis risk factors. A potential limitation of our study is that, because we were comparing single racial subgroups to each other (e.g., Black vs. White women), analysis was necessarily limited to those who reported only a single race. Future studies need to be performed to analyze the impact of clinical outcomes following MOF in multiracial women. Also, compared to White women, there were markedly fewer women who self-reported being American/Indian/Alaskan Native and Native Hawaiian/Pacific Islander, leading to a small sample size for these racial groups. Similarly, compared to non-Hispanic women, fewer women self-reported being Hispanic, limiting the generalizability of our study. Because this was an observational study, our results may be limited by residual confounding, despite controlling for potential confounders in our analysis. For example, the time between assessment of physical function and MOF event varied but on average was about 7 years before the event and thus may not accurately capture physical function at the time of the MOF event. Additionally, low socioeconomic status is a risk factor for mortality,^21^ and detailed information regarding income that was not available in our dataset may be a confounder of these findings.

In addition, confirming that mortality is due to MOF is difficult. Currently, there is no clear explanation of what drives the fracture-mortality association. Some studies suggest that the fracture-mortality association is related to underlying health and comorbidities rather than the fracture event,^22,23^ but other studies have not found such an association.^13^ Moreover, 5 years following MOF, it is even less certain that mortality can be attributed to MOF; and therefore, our 5-year mortality results should be interpreted with caution. Finally, another limitation of our study is the lack of body composition and bone mineral density (BMD) data, which may impact fracture risk and mortality.^14,15^ Future studies incorporating BMD assessments are needed to further examine racial differences in bone health and post-fracture outcomes.

Beyond these clinical factors, it is important to recognize that race and ethnicity are social constructs which serve as proxies for implicit bias in medical decision-making.^24^ Therefore, differences in mortality following MOF in this analysis may be due to disparities in healthcare access or implicit bias. These findings are not to be interpreted as suggesting higher innate susceptibility to mortality following MOF in racial and ethnic subgroups.^25,26^

## CONCLUSIONS

This large prospective cohort study found that mortality following MOF differed by race and ethnicity. Compared to White women, the risk of mortality 1 year following MOF was higher among Black women and lower among Asian women. Additionally, 5 years following MOF mortality was higher among American Indian/Alaska Natives (and lower among Asian women) compared to White women and lower among Hispanic/Latina compared to non-Hispanic Latina women. These findings are a first step toward understanding clinical outcomes following MOF and highlight potential opportunities for improvement in current clinical approaches. Future research is needed to delineate the mechanism behind these associations to generate efforts to improve outcomes following MOF across racial groups.

## Supplementary Information

Below is the link to the electronic supplementary material.

Supplementary file1 (DOCX 52.3 KB)

Supplementary file2 (DOCX 424 KB)

## Data Availability

See Data Sharing Supplement.

## Declarations:

### Disclaimer:

Representatives of the National Heart, Lung, and Blood Institute had a role in the design and conduct of this study; collection, management, and analysis of the data; and approval of the manuscript. They had no role in the interpretation of the data, preparation of the manuscript, decision to submit the manuscript for publication, or review of the manuscript.

## References

[1] **Kanis JA, Melton LJ 3rd, Christiansen C, Johnston CC, Khaltaev N.** The diagnosis of osteoporosis. J Bone Miner Res. 1994;9(8):1137-1141. 10.1002/jbmr.5650090802. 7976495 10.1002/jbmr.5650090802

[2] **Port L, Center J, Briffa NK, Nguyen T, Cumming R, Eisman J.** Osteoporotic fracture: missed opportunity for intervention. Osteoporos Int. 2003;14(9):780-784. 10.1007/s00198-003-1452-x. 12904835 10.1007/s00198-003-1452-x

[3] **Bliuc D, Nguyen ND, Milch VE, Nguyen TV, Eisman JA, Center JR.** Mortality risk associated with low-trauma osteoporotic fracture and subsequent fracture in men and women. JAMA. 2009;301(5):513-521. 10.1001/jama.2009.50. 19190316 10.1001/jama.2009.50

[4] **Cooper C.** The crippling consequences of fractures and their impact on quality of life. Am J Med. 1997;103(2A):12S-17S9302893. 9302893 10.1016/s0002-9343(97)90022-x

[5] **Randell AG, Nguyen TV, Bhalerao N, Silverman SL, Sambrook PN, Eisman JA.** Deterioration in quality of life following hip fracture: a prospective study. Osteoporos Int. 2000;11(5):460-466 10912850 10912850 10.1007/s001980070115

[6] **Center JR, Nguyen TV, Schneider P, Sambrook PN, Eisman JA.** Mortality after all major types of osteoporotic fracture in men and women: an observational study. Lancet. 1999;353(9156):878-88210093980. 10093980 10.1016/S0140-6736(98)09075-8

[7] **Wright NC, Chen L, Saag KG, Brown CJ, Shikany JM, Curtis JR.** Racial disparities exist in outcomes after major fragility fractures. J Am Geriatr Soc. 2020;68(8):1803-1810. 10.1111/jgs.16455. 32337717 10.1111/jgs.16455 PMC7935465

[8] Design of the Women's Health Initiative clinical trial and observational study. The Women's Health Initiative Study Group. Control Clin Trials. 1998;19(1):61–109. 10.1016/s0197-2456(97)00078-0 10.1016/s0197-2456(97)00078-0 9492970

[9] **Stefanick M, Aragaki A, Cené C, Dilworth-Anderson P, Follis S, Garcia L, Jiménez M, Koop C.** WHI Race and Ethnicity Language and Data Application and Interpretation Guide. 2021. whi.org. https://www.whi.org/doc/WHI-Race-and-Ethnicity-Language-and-Data-Interpretation-Guide.pdf. Accessed 18 Apr 2025.

[10] **Garcia L, Follis S, Thomson CA, et al.** Correction: Taking action to advance the study of race and ethnicity: the Women's Health Initiative (WHI). Womens Midlife Health. 2022;8(1):13. Published 2022 Nov 25. 10.1186/s40695-022-00083-w. 36434684 10.1186/s40695-022-00083-w PMC9700984

[11] **Andresen EM, Bowley N, Rothenberg BM, Panzer R, Katz P.** Test-retest performance of a mailed version of the Medical Outcomes Study 36-Item Short-Form Health Survey among older adults. Med Care. 1996;34(12):1165-1170. 10.1097/00005650-199612000-00001. 8962582 10.1097/00005650-199612000-00001

[12] **Center JR, Nguyen TV, Schneider P, Sambrook PN, Eisman JA.** Mortality after all major types of osteoporotic fracture in men and women: an observational study. Lancet. 1999;353(9156):878-88210093980. 10093980 10.1016/S0140-6736(98)09075-8

[13] **Cauley JA, Thompson DE, Ensrud KC, Scott JC, Black D.** Risk of mortality following clinical fractures. Osteoporos Int. 2000;11(7):556-561. 10.1007/s001980070075. 11069188 10.1007/s001980070075

[14] **Bliuc D, Alarkawi D, Nguyen TV, Eisman JA, Center JR.** Risk of subsequent fractures and mortality in elderly women and men with fragility fractures with and without osteoporotic bone density: the Dubbo Osteoporosis Epidemiology Study. J Bone Miner Res. 2015;30(4):637-46. 10.1002/jbmr.2393. 25359586 10.1002/jbmr.2393

[15] **Bliuc D, Nguyen ND, Milch VE, Nguyen TV, Eisman JA, Center JR.** Mortality risk associated with low-trauma osteoporotic fracture and subsequent fracture in men and women. JAMA. 2009;301(5):513-21. 10.1001/jama.2009.50. 19190316 10.1001/jama.2009.50

[16] **Neuner JM, Zhang X, Sparapani R, Laud PW, Nattinger AB.** Racial and socioeconomic disparities in bone density testing before and after hip fracture. J Gen Intern Med. 2007;22(9):1239-45. 10.1007/s11606-007-0217-1. 17594131 10.1007/s11606-007-0217-1 PMC2219762

[17] **Miller RG, Ashar BH, Cohen J, Camp M, Coombs C, Johnson E, Schneyer CR.** Disparities in osteoporosis screening between at-risk African-American and white women. J Gen Intern Med. 2005;20(9):847-51. 10.1111/j.1525-1497.2005.0157.x. 16117754 10.1111/j.1525-1497.2005.0157.x PMC1490213

[18] **Film R, Fritz J, Adams T, Johnson A, Sun N, Falvey J.** Racial disparities in outpatient physical therapy use after hip fracture: a retrospective cohort study. J Orthop Sports Phys Ther. 2024;54(12):776-782. 10.2519/jospt.2024.12641. 39602204 10.2519/jospt.2024.12641 PMC11900720

[19] **Cooper C, Atkinson EJ, Jacobsen SJ, O'Fallon WM, Melton LJ 3rd.** Population-based study of survival after osteoporotic fractures. Am J Epidemiol. 1993;137(9):1001-1005. 10.1093/oxfordjournals.aje.a116756. 8317445 10.1093/oxfordjournals.aje.a116756

[20] **Rizkallah M, Bachour F, Khoury ME, et al.** Comparison of morbidity and mortality of hip and vertebral fragility fractures: which one has the highest burden?. Osteoporos Sarcopenia. 2020;6(3):146-150. 10.1016/j.afos.2020.07.002. 33102809 10.1016/j.afos.2020.07.002 PMC7573502

[21] **Lantz PM, House JS, Lepkowski JM, Williams DR, Mero RP, Chen J.** Socioeconomic factors, health behaviors, and mortality: results from a nationally representative prospective study of US adults. JAMA. 1998;279(21):1703-1708. 10.1001/jama.279.21.1703. 9624022 10.1001/jama.279.21.1703

[22] **Browner WS, Pressman AR, Nevitt MC, Cummings SR.** Mortality following fractures in older women: the study of osteoporotic fractures. Arch Intern Med. 1996;156(14):1521-15258687260. 8687260

[23] **Tosteson AN, Gottlieb DJ, Radley DC, Fisher ES, Melton LJ III.** Excess mortality following hip fracture: the role of underlying health status. Osteoporos Int. 2007;18(11):1463-147217726622. 17726622 10.1007/s00198-007-0429-6 PMC2729704

[24] **Boyd RW, Lindo EG, Weeks LD, McLemore MR.** On racism: a new standard for publishing on racial health inequities. Health Affairs Blog. Published online July 2, 2020. 10.1377/forefront.20200630.939347.

[25] **Deyrup A, Graves JL Jr.** Racial biology and medical misconceptions. N Engl J Med. 2022;386(6):501-503. 10.1056/NEJMp2116224 35119803 10.1056/NEJMp2116224

[26] **Wright JL, Freed GL, Hendricks-Muñoz KD, et al.** Committee on diversity, inclusion and equity on behalf of the American Pediatric Society. Achieving equity through science and integrity: dismantling race-based medicine. Pediatr Res. 2022;91(7):1641-1644. 10.1038/s41390-022-02041-8 35383261 10.1038/s41390-022-02041-8

