# Supplementary material for: Race, Ethnicity, and Mortality Following Major Osteoporotic Fracture: Results from the Women’s Health Initiative Study
Source: J Gen Intern Med. 2025 Apr 24;40(11):2583–92. doi: 10.1007/s11606-025-09506-6 (PMC12405089; doi:10.1007/s11606-025-09506-6)
Supplement: Supplementary file 1 — Supplementary file1 (DOCX 52.3 KB) [file 11606_2025_9506_MOESM1_ESM.docx]

Race, Ethnicity, and Mortality Following Major Osteoporotic Fracture: Results from the Women’s Health Initiative Study

Michaela Juels BS, Joseph C. Larson MS., Kristine E. Ensrud, MD, MPH, Marcia L. Stefanick Ph.D., Aladdin H. Shadyab Ph.D., Lorena Garcia MPH, DrPH, Rami Nassir Ph.D., Peter F. Schnatz DO, FACOG, FACP, NCMP, Rebecca Nelson Ph.D., Carolyn J. Crandall, MD, MS, MACP

Author Affiliations:

Michaela Juels, BS: David Geffen School of Medicine, University of California, Los Angeles, USA.

Joseph C. Larson, MS: Fred Hutchinson Cancer Research Center, Seattle, WA, USA

Email: jlarson@whi.org

Kristine E. Ensrud, MD, MPH: Division of Epidemiology and Community Health and Department of Medicine, University of Minnesota, Minneapolis MN, USA.

Email: ensru001@umn.edu

Marcia L. Stefanick, PhD: Departments of Medicine (Stanford Prevention Research Center) and of Obstetrics & Gynecology, Stanford University, Stanford CA, USA.

Email: stefanick@stanford.edu

Aladdin H. Shadyab Ph.D: Herbert Wertheim School of Public Health and Human Longevity Science and Division of Geriatrics, Gerontology, and Palliative Care, Department of Medicine, University of California San Diego, La Jolla, CA. USA.

Email: ahshadya@health.ucsd.edu

Lorena Garcia, MPH, DrPH: Department of Public Health Sciences, School of Medicine, University of California, Davis, CA, USA.

Email: [lgarcia@ucdavis.edu](mailto:lgarcia@ucdavis.edu)

Rami Nassir Ph.D: Department of Pathology, School of Medicine, Umm Al-Qura University, Mecca, Saudi Arabia

Email: rmnassir@formerstudents.ucdavis.edu

Peter F. Schnatz, DO: Reading Hospital / Tower Health & Drexel University; Departments of Obstetrics, Gynecology and Internal Medicine, Philadelphia, PA. USA.

Email: peter.schnatz@towerhealth.org

Rebecca A. Nelson, PhD: City of Hope Comprehensive Cancer Center, Department of Computational and Quantitative Medicine, Division of Biostatistics, Duarte, CA. USA.

Email: rnelson@coh.org

Carolyn J. Crandall, MD, MS, MACP: Division of General Internal Medicine and Health Services Research, Dept. of Medicine, David Geffen School of Medicine at University of California, Los Angeles, USA. Email [ccrandall@mednet.ucla.edu](mailto:ccrandall@mednet.ucla.edu)

Corresponding Author: Michaela Juels

David Geffen School of Medicine at UCLA
Student Affairs Office
Geffen Hall, Suite 200 
885 Tiverton Drive, Box 951720
Los Angeles, CA, USA 90095-1720

[mjuels@mednet.ucla.edu](mailto:mjuels@mednet.ucla.edu)

Supplement 1

eTable 1. Characteristics of Participants Overall and by 5-year mortality (n=29506)

eTable 2. Key Characteristics by Ethnicity (n=32675)

eTable 3: Key Characteristics at time of Major Osteoporotic Fracture event by Race (n=32675)

eTable 4: 1-year mortality after MOF by Ethnicity

eTable 5: Associations between type of major osteoporotic fracture and 1-year mortality

eTable 6 Associations between type of major osteoporotic fracture and 5-year mortality

eTable 1. Characteristics of Participants Overall and by 5-year mortality (n=29506)

|  | All Participants (n=29506) | | 5-Year Mortality | | | | | | |
| --- | --- | --- | --- | --- | --- | --- | --- | --- | --- |
|  |  |  | No (n=22978) | | Yes (n=6528) | |  | |  |
| Demographic | n | % | n | % | n | % | p-value |  |  |
| Age, years, mean (SD) | 76.3 | (8.5) | 74.5 | (7.9) | 82.6 | (7.6) | <0.001 |  |  |
| <70 | 6349 | 21.5 | 5981 | 26.0 | 368 | 5.6 |  |  |  |
| 70 – 80 | 13566 | 46.0 | 11608 | 50.5 | 1958 | 30.0 |  |  |  |
| >80 | 9591 | 32.5 | 5389 | 23.5 | 4202 | 64.4 |  |  |  |
| Race |  |  |  |  |  |  | <0.001 |  |  |
| American Indian / Alaska Native | 45 | 0.2 | 28 | 0.1 | 17 | 0.3 |  |  |  |
| Asian | 388 | 1.3 | 332 | 1.4 | 56 | 0.9 |  |  |  |
| Native Hawaiian / Pacific Islander | 9 | 0.0 | 8 | 0.0 | 1 | 0.0 |  |  |  |
| Black / African American | 852 | 2.9 | 688 | 3.0 | 164 | 2.5 |  |  |  |
| White | 28212 | 95.6 | 21922 | 95.4 | 6290 | 96.4 |  |  |  |
| Ethnicity |  |  |  |  |  |  | <0.001 |  |  |
| Not Hispanic / Latina | 28926 | 98.0 | 22489 | 97.9 | 6437 | 98.6 |  |  |  |
| Hispanic / Latina | 580 | 2.0 | 489 | 2.1 | 91 | 1.4 |  |  |  |
| WHI Study Component |  |  |  |  |  |  | <0.001 |  |  |
| Clinical Trial | 12809 | 43.4 | 10104 | 44.0 | 2705 | 41.4 |  |  |  |
| Observational Study | 16697 | 56.6 | 12874 | 56.0 | 3823 | 58.6 |  |  |  |
| MOF Fracture Site^[[1]](#footnote-1)^ |  |  |  |  |  |  |  |  |  |
| Hip | 5483 | 18.6 | 3345 | 14.6 | 2138 | 32.8 | <0.001 |  |  |
| Spine | 7758 | 26.3 | 5748 | 25.0 | 2010 | 30.8 | <0.001 |  |  |
| Upper arm / shoulder | 5860 | 19.9 | 4724 | 20.6 | 1136 | 17.4 | <0.001 |  |  |
| Lower arm / wrist | 10984 | 37.2 | 9569 | 41.6 | 1415 | 21.7 | <0.001 |  |  |
| Medical History |  |  |  |  |  |  |  |  |  |
| Cancer | 6342 | 21.5 | 4255 | 18.5 | 2087 | 32.0 | <0.001 |  |  |
| CVD | 3868 | 13.1 | 2267 | 9.9 | 1601 | 24.5 | <0.001 |  |  |
| MI | 1597 | 5.4 | 953 | 4.1 | 644 | 9.9 | <0.001 |  |  |
| CABG | 782 | 2.7 | 467 | 2.0 | 315 | 4.8 | <0.001 |  |  |
| PTCA | 1416 | 4.8 | 862 | 3.8 | 554 | 8.5 | <0.001 |  |  |
| Stroke | 1494 | 5.1 | 771 | 3.4 | 723 | 11.1 | <0.001 |  |  |
| COPD | 3796 | 12.9 | 2628 | 11.4 | 1168 | 17.9 | <0.001 |  |  |
| Treated diabetes | 3878 | 13.1 | 2580 | 11.2 | 1298 | 19.9 | <0.001 |  |  |
| Education |  |  |  |  |  |  | <0.001 |  |  |
| ≤ High school / GED | 5919 | 20.1 | 4533 | 19.7 | 1386 | 21.2 |  |  |  |
| School after high school | 11080 | 37.6 | 8559 | 37.2 | 2521 | 38.6 |  |  |  |
| College degree or higher | 12507 | 42.4 | 9886 | 43.0 | 2621 | 40.2 |  |  |  |
| Region |  |  |  |  |  |  | <0.001 |  |  |
| Northeast | 7646 | 25.9 | 5983 | 26.0 | 1663 | 25.5 |  |  |  |
| South | 6781 | 23.0 | 5323 | 23.2 | 1458 | 22.3 |  |  |  |
| Midwest | 6704 | 22.7 | 5302 | 23.1 | 1402 | 21.5 |  |  |  |
| West | 8375 | 28.4 | 6370 | 27.7 | 2005 | 30.7 |  |  |  |
| BMI, kg/m^2^, mean (SD) | 27.6 | (5.7) | 27.6 | (5.7) | 27.4 | (5.8) | <0.001 |  |  |
| Physical function^[[2]](#footnote-2)^ (0-100), mean (SD) | 74.6 | (23.5) | 77.0 | (22.1) | 65.9 | (26.1) | <0.001 |  |  |
| Emotional well-being^b^ (0-100), mean (SD) | 78.7 | (14.7) | 78.9 | (14.5) | 78.0 | (15.0) | <0.001 |  |  |
| Smoking |  |  |  |  |  |  | <0.001 |  |  |
| Never | 14981 | 50.8 | 11860 | 51.6 | 3121 | 47.8 |  |  |  |
| Past | 13316 | 45.1 | 10219 | 44.5 | 3097 | 47.4 |  |  |  |
| Current | 1209 | 4.1 | 899 | 3.9 | 310 | 4.7 |  |  |  |
| Alcohol Use |  |  |  |  |  |  | <0.001 |  |  |
| Never | 2832 | 9.6 | 2219 | 9.7 | 613 | 9.4 |  |  |  |
| Past | 5782 | 19.6 | 4211 | 18.3 | 1571 | 24.1 |  |  |  |
| Current | 20892 | 70.8 | 16548 | 72.0 | 4344 | 66.5 |  |  |  |
| Medication use |  |  |  |  |  |  |  |  |  |
| Osteoporosis Medications | 8155 | 27.6 | 6298 | 27.4 | 1857 | 28.4 | 0.10 |  |  |
| Bisphosphonate | 6748 | 22.9 | 5198 | 22.6 | 1550 | 23.7 | 0.06 |  |  |
| Calcitonin | 884 | 3.0 | 665 | 2.9 | 219 | 3.4 | 0.05 |  |  |
| Parathyroid hormone | 54 | 0.2 | 46 | 0.2 | 8 | 0.1 | 0.20 |  |  |
| SERMs | 1247 | 4.2 | 986 | 4.3 | 261 | 4.0 | 0.30 |  |  |
| Rank Ligand Inhibitors | 3 | 0.0 | 1 | 0.0 | 2 | 0.0 | 0.06 |  |  |

eTable 2. Key Characteristics by Ethnicity (n=32675)

|  | Ethnicity | | | | |
| --- | --- | --- | --- | --- | --- |
|  | Not  Hispanic / Latina (n=32015) | | | Hispanic / Latina  (n=660) | |
| Demographic | n | % | n | | % |
| Age, years, mean (SD) | 77.0 | (8.5) | 75.3 | | (9.2) |
| <70 | 6168 | 19.3 | 181 | | 27.4 |
| 70 – 80 | 14325 | 44.7 | 273 | | 41.4 |
| >80 | 11522 | 36.0 | 206 | | 31.2 |
| Race |  |  |  | |  |
| American Indian / Alaska Native | 46 | 0.1 | 7 | | 1.1 |
| Asian | 438 | 1.4 | 8 | | 1.2 |
| Native Hawaiian / Pacific Islander | 10 | 0.0 | 1 | | 0.2 |
| Black / African American | 927 | 2.9 | 15 | | 2.3 |
| White | 30594 | 95.6 | 629 | | 95.3 |
| WHI Study Component |  |  |  | |  |
| Clinical Trial | 13861 | 43.3 | 337 | | 51.1 |
| Observational Study | 18154 | 56.7 | 323 | | 48.9 |
| MOF Fracture Site^[[3]](#footnote-3)^ |  |  |  | |  |
| Hip | 6184 | 19.3 | 64 | | 9.7 |
| Spine | 8457 | 26.4 | 155 | | 23.5 |
| Upper arm / shoulder | 6386 | 19.9 | 181 | | 27.4 |
| Lower arm / wrist | 11656 | 36.4 | 272 | | 41.2 |
| Medical History |  |  |  | |  |
| Cancer | 7040 | 22.0 | 109 | | 16.5 |
| CVD | 4249 | 13.3 | 85 | | 12.9 |
| MI | 1721 | 5.4 | 31 | | 4.7 |
| CABG | 822 | 2.6 | 22 | | 3.3 |
| PTCA | 1596 | 5.0 | 25 | | 3.8 |
| Stroke | 1667 | 5.2 | 33 | | 5.0 |
| COPD | 4288 | 13.4 | 86 | | 13.0 |
| Treated diabetes | 4405 | 13.8 | 137 | | 20.8 |
| Education |  |  |  | |  |
| ≤ High school / GED | 6233 | 19.5 | 191 | | 28.9 |
| School after high school | 11918 | 37.2 | 287 | | 43.5 |
| College degree or higher | 13864 | 43.3 | 182 | | 27.6 |
| Region |  |  |  | |  |
| Northeast | 8335 | 26.0 | 89 | | 13.5 |
| South | 7280 | 22.7 | 251 | | 38.0 |
| Midwest | 7388 | 23.1 | 35 | | 5.3 |
| West | 9012 | 28.1 | 285 | | 43.2 |
| BMI, kg/m^2^, mean (SD) | 27.5 | (5.7) | 28.8 | | (5.9) |
| Physical function^a^ (0-100), mean (SD) | 74.6 | (23.3) | 74.0 | | (23.9) |
| Emotional well-being^[[4]](#footnote-4)^ (0-100), mean (SD) | 78.9 | (14.5) | 74.8 | | (17.3) |
| Smoking |  |  |  | |  |
| Never | 16265 | 50.8 | 389 | | 58.9 |
| Past | 14507 | 45.3 | 249 | | 37.7 |
| Current | 1243 | 3.9 | 22 | | 3.3 |
| Alcohol Use |  |  |  | |  |
| Never | 2996 | 9.4 | 82 | | 12.4 |
| Past | 6229 | 19.5 | 153 | | 23.2 |
| Current | 22790 | 71.2 | 425 | | 64.4 |
| Medication use |  |  |  | |  |
| Osteoporosis Medications | 9051 | 28.3 | 153 | | 23.2 |
| Bisphosphonates | 7495 | 23.4 | 127 | | 19.2 |
| Calcitonins | 938 | 2.9 | 25 | | 3.8 |
| Parathyroid hormones | 64 | 0.2 | 0 | | 0.0 |
| SERMs | 1398 | 4.4 | 22 | | 3.3 |
| Rank Ligand Inhibitors | 3 | 0.0 | 1 | | 0.2 |

eTable 3: Key Characteristics at time of Major Osteoporotic Fracture event by Race (n=32675)

|  | Race | | | | | | | | | |
| --- | --- | --- | --- | --- | --- | --- | --- | --- | --- | --- |
|  | American Indian / Alaska Native (n=53) | | Asian  (n=446) | | Native Hawaiian / Pacific Islander (n=11) | | Black / African American (n=942) | | White (n=31223) | |
| Demographic | n | % | n | % | n | % | n | % | n | % |
| Age, years, mean (SD) | 75.6 | (8.5) | 77.3 | (8.7) | 70.7 | (8.6) | 74.2 | (9.3) | 77.1 | (8.5) |
| <70 | 12 | 22.6 | 85 | 19.1 | 6 | 54.5 | 291 | 30.9 | 5955 | 19.1 |
| 70 – 80 | 26 | 49.1 | 205 | 46.0 | 4 | 36.4 | 402 | 42.7 | 13961 | 44.7 |
| >80 | 15 | 28.3 | 156 | 35.0 | 1 | 9.1 | 249 | 26.4 | 11307 | 36.2 |
| Ethnicity |  |  |  |  |  |  |  |  |  |  |
| Not Hispanic / Latina | 46 | 86.8 | 438 | 98.2 | 10 | 90.9 | 927 | 98.4 | 30594 | 98.0 |
| Hispanic / Latina | 7 | 13.2 | 8 | 1.8 | 1 | 9.1 | 15 | 1.6 | 629 | 2.0 |
| WHI Study Component |  |  |  |  |  |  |  |  |  |  |
| Clinical Trial | 25 | 47.2 | 187 | 41.9 | 9 | 81.8 | 510 | 54.1 | 13467 | 43.1 |
| Observational Study | 28 | 52.8 | 259 | 58.1 | 2 | 18.2 | 432 | 45.9 | 17756 | 56.9 |
| MOF Fracture Site^[[5]](#footnote-5)^ |  |  |  |  |  |  |  |  |  |  |
| Hip | 6 | 11.3 | 66 | 14.8 | 1 | 9.1 | 128 | 13.6 | 6047 | 19.4 |
| Spine | 12 | 22.6 | 136 | 30.5 | 1 | 9.1 | 165 | 17.5 | 8298 | 26.6 |
| Upper arm / shoulder | 8 | 15.1 | 76 | 17.0 | 4 | 36.4 | 253 | 26.9 | 6226 | 19.9 |
| Lower arm / wrist | 29 | 54.7 | 176 | 39.5 | 5 | 45.5 | 420 | 44.6 | 11298 | 36.2 |
| Medical History |  |  |  |  |  |  |  |  |  |  |
| Cancer | 13 | 24.5 | 74 | 16.6 | 3 | 27.3 | 148 | 15.7 | 6911 | 22.1 |
| CVD | 8 | 15.1 | 40 | 9.0 | 0 | 0.0 | 155 | 16.5 | 4131 | 13.2 |
| MI | 3 | 5.7 | 12 | 2.7 | 0 | 0.0 | 66 | 7.0 | 1671 | 5.4 |
| CABG | 2 | 3.8 | 8 | 1.8 | 0 | 0.0 | 29 | 3.1 | 805 | 2.6 |
| PTCA | 2 | 3.8 | 8 | 1.8 | 0 | 0.0 | 40 | 4.2 | 1571 | 5.0 |
| Stroke | 4 | 7.5 | 20 | 4.5 | 0 | 0.0 | 76 | 8.1 | 1600 | 5.1 |
| COPD | 12 | 22.6 | 52 | 11.7 | 1 | 9.1 | 140 | 14.9 | 4169 | 13.4 |
| Treated diabetes | 18 | 34.0 | 87 | 19.5 | 3 | 27.3 | 277 | 29.4 | 4157 | 13.3 |
| Education |  |  |  |  |  |  |  |  |  |  |
| ≤ High school / GED | 8 | 15.1 | 71 | 15.9 | 2 | 18.2 | 212 | 22.5 | 6131 | 19.6 |
| School after high school | 30 | 56.6 | 146 | 32.7 | 7 | 63.6 | 347 | 36.8 | 11675 | 37.4 |
| College degree or higher | 15 | 28.3 | 229 | 51.3 | 2 | 18.2 | 383 | 40.7 | 13417 | 43.0 |
| Region |  |  |  |  |  |  |  |  |  |  |
| Northeast | 8 | 15.1 | 24 | 5.4 | 0 | 0.0 | 168 | 17.8 | 8224 | 26.3 |
| South | 16 | 30.2 | 21 | 4.7 | 1 | 9.1 | 429 | 45.5 | 7064 | 22.6 |
| Midwest | 5 | 9.4 | 26 | 5.8 | 0 | 0.0 | 241 | 25.6 | 7151 | 22.9 |
| West | 24 | 45.3 | 375 | 84.1 | 10 | 90.9 | 104 | 11.0 | 8784 | 28.1 |
| BMI, kg/m^2^, mean (SD) | 31.7 | (7.5) | 24.5 | (4.5) | 30.2 | (7.5) | 30.6 | (6.7) | 27.5 | (5.6) |
| Physical function^[[6]](#footnote-6)^ (0-100), mean (SD) | 60.9 | (29.7) | 78.2 | (22.2) | 87.3 | (11.9) | 68.7 | (26.4) | 74.8 | (23.2) |
| Emotional well-being^b^ (0-100), mean (SD) | 77.1 | (17.6) | 80.8 | (12.9) | 82.6 | (14.6) | 76.5 | (16.5) | 78.9 | (14.5) |
| Smoking |  |  |  |  |  |  |  |  |  |  |
| Never | 24 | 45.3 | 321 | 72.0 | 2 | 18.2 | 439 | 46.6 | 15868 | 50.8 |
| Past | 26 | 49.1 | 119 | 26.7 | 9 | 81.8 | 433 | 46.0 | 14169 | 45.4 |
| Current | 3 | 5.7 | 6 | 1.3 | 0 | 0.0 | 70 | 7.4 | 1186 | 3.8 |
| Alcohol Use |  |  |  |  |  |  |  |  |  |  |
| Never | 5 | 9.4 | 164 | 36.8 | 3 | 27.3 | 163 | 17.3 | 2743 | 8.8 |
| Past | 18 | 34.0 | 113 | 25.3 | 2 | 18.2 | 307 | 32.6 | 5942 | 19.0 |
| Current | 30 | 56.6 | 169 | 37.9 | 6 | 54.5 | 472 | 50.1 | 22538 | 72.2 |
| Medication use |  |  |  |  |  |  |  |  |  |  |
| Osteoporosis Medications | 14 | 26.4 | 186 | 41.7 | 1 | 9.1 | 143 | 15.2 | 8860 | 28.4 |
| Bisphosphonate | 11 | 20.8 | 154 | 34.5 | 1 | 9.1 | 114 | 12.1 | 7342 | 23.5 |
| Calcitonin | 0 | 0.0 | 24 | 5.4 | 0 | 0.0 | 9 | 1.0 | 930 | 3.0 |
| Parathyroid hormone | 0 | 0.0 | 1 | 0.2 | 0 | 0.0 | 2 | 0.2 | 61 | 0.2 |
| SERMs | 4 | 7.5 | 25 | 5.6 | 0 | 0.0 | 25 | 2.7 | 1366 | 4.4 |
| Rank Ligand Inhibitors | 0 | 0.0 | 0 | 0.0 | 0 | 0.0 | 1 | 0.1 | 3 | 0.0 |

eTable 4: 1-year mortality after MOF by Ethnicity

|  | Ethnicity | |  |
| --- | --- | --- | --- |
|  | Not Hispanic / Latina | Hispanic / Latina |  |
| n | 32015 | 660 |  |
| Events | 1680 | 25 |  |
| % | 5.2 | 3.8 |  |
| Model^[[7]](#footnote-7)^ | OR (95% CI) | OR (95% CI) | p-value |
| Model 1 | 1.00 (ref) | 0.86 (0.57, 1.30) | 0.47 |
| Model 2 | 1.00 (ref) | 0.85 (0.56, 1.30) | 0.46 |
| Model 3 | 1.00 (ref) | 0.85 (0.56, 1.29) | 0.45 |
| Model 4 | 1.00 (ref) | 0.84 (0.55, 1.28) | 0.43 |

eTable 5: Associations between type of major osteoporotic fracture and 1-year mortality

| MOF Site | OR (95% CI)^[[8]](#footnote-8)^ |
| --- | --- |
| Lower arm / wrist | 1.00 (ref) |
| Upper arm / shoulder | 1.37 (1.15, 1.64) |
| Spine | 1.88 (1.60, 2.19) |
| Hip | 2.79 (2.40, 3.25) |

eTable 6 Associations between type of major osteoporotic fracture and 5-year mortality

| MOF Site | OR (95% CI)^[[9]](#footnote-9)^ |
| --- | --- |
| Lower arm / wrist | 1.00 (ref) |
| Upper arm / shoulder | 1.21 (1.10, 1.34) |
| Spine | 1.68 (1.54, 1.83) |
| Hip | 2.24 (2.05, 2.45) |

1. 562 participants had fractures at multiple sites. [↑](#footnote-ref-1)
2. Assessed using the RAND SF-36 survey.

   Abbreviations: MOF, major osteoporotic fracture; CVD, cardiovascular disease; MI, myocardial infarction; CABG, coronary artery bypass graft; PTCA, Percutaneous transluminal coronary angioplasty; COPD, chronic obstructive pulmonary disease; GED, general educational development; BMI, body mass index; SERMs, Selective estrogen receptor modulator; RAND SF-36, 36-Item Short Form Health Survey; WHI, Women’s Health Initiative.

   Mean (SD) years from covariate assessment to MOF fracture: Age, 0.0 (0.0); Race/Ethnicity, 0.6 (2.7); medical history except COPD, 0.1 (0.2); COPD 4.3 (4.7); education, region 12.1 (6.8); BMI 8.0 (6.2); physical function 6.7 (4.7); emotional well-being 6.6 (4.6); smoking 3.5 (3.2); alcohol use 7.1 (4.7); medication use 2.8 (3.6); [↑](#footnote-ref-2)
3. Abbreviations: MOF, major osteoporotic fracture; CVD, cardiovascular disease; MI, myocardial infarction; CABG, coronary artery bypass graft; PTCA, Percutaneous transluminal coronary angioplasty; COPD, chronic obstructive pulmonary disease; GED, general educational development; BMI, body mass index; SERMs, Selective estrogen receptor modulator; RAND SF-36, 36-Item Short Form Health Survey; WHI, Women’s Health Initiative.

   660 participants had fractures at multiple sites. [↑](#footnote-ref-3)
4. Abbreviations: MOF, major osteoporotic fracture; CVD, cardiovascular disease; MI, myocardial infarction; CABG, coronary artery bypass graft; PTCA, Percutaneous transluminal coronary angioplasty; COPD, chronic obstructive pulmonary disease; GED, general educational development; BMI, body mass index; SERMs, Selective estrogen receptor modulator; RAND SF-36, 36-Item Short Form Health Survey; WHI, Women’s Health Initiative.

   Assessed using the RAND SF-36 survey. [↑](#footnote-ref-4)
5. 660 participants had fractures at multiple sites. [↑](#footnote-ref-5)
6. Assessed using the RAND SF-36 survey.

   Abbreviations: MOF, major osteoporotic fracture; CVD, cardiovascular disease; MI, myocardial infarction; CABG, coronary artery bypass graft; PTCA, Percutaneous transluminal coronary angioplasty; COPD, chronic obstructive pulmonary disease; GED, general educational development; BMI, body mass index; SERMs, Selective estrogen receptor modulator; RAND SF-36, 36-Item Short Form Health Survey; WHI, Women’s Health Initiative.

   Mean (SD) years from covariate assessment to MOF fracture:

   Age, 0.0 (0.0); Race/Ethnicity, 0.6 (2.7); medical history except COPD, 0.1 (0.2); COPD 4.3 (4.7); education, region 12.1 (6.8); BMI 8.0 (6.2); physical function 6.7 (4.7); emotional well-being 6.6 (4.6); smoking 3.5 (3.2); alcohol use 7.1 (4.7); medication use 2.8 (3.6). [↑](#footnote-ref-6)
7. All logistic regression models are adjusted for race.

   Model 1: Adjusted for fracture site, WHI study component, age, education, and region

   Model 2: Model 1 + History of cancer, history of cardiovascular disease, history of chronic obstructive pulmonary disease, history of treated diabetes mellitus

   Model 3: Model 2 + current smoking, current alcohol use, body mass index, physical function score, emotional well-being score

   Model 4: Model 4 + ever-used osteoporosis medication

   Abbreviations: OR, Odds ratio; CI, confidence interval; WHI, Women’s Health Initiative. [↑](#footnote-ref-7)
8. All models are adjusted for race, fracture site, WHI study component, age, education, and region, history of cancer, history of cardiovascular disease, history of chronic obstructive pulmonary disease, history of lupus, history of treated diabetes, smoking, alcohol, body mass index, physical function^b^, emotional well-being^b^, and osteoporosis medication use

   ^b^ Assessed using the RAND SF-36 survey, 36-Item Short Form Health Survey.

   Abbreviations: MOF, major osteoporotic fracture, OR, Odds ratio; CI, confidence interval; WHI, Women’s Health Initiative. [↑](#footnote-ref-8)
9. All models are adjusted for race, fracture site, WHI study component, age, education, and region, history of cancer, history of cardiovascular disease, history of chronic obstructive pulmonary disease, history of lupus, history of treated diabetes, smoking, alcohol, body mass index, physical function^b^, emotional well-being^b^, and osteoporosis medication use

   ^b^ Assessed using the RAND SF-36 survey, 36-Item Short Form Health Survey.

   Abbreviations: MOF, major osteoporotic fracture, OR, Odds ratio; CI, confidence interval; WHI, Women’s Health Initiative. [↑](#footnote-ref-9)
