# Supplementary material for: Race, Ethnicity, and Mortality Following Major Osteoporotic Fracture: Results from the Women’s Health Initiative Study
Source: J Gen Intern Med. 2025 Apr 24;40(11):2583–92. doi: 10.1007/s11606-025-09506-6 (PMC12405089; doi:10.1007/s11606-025-09506-6)

Race, Ethnicity, and Mortality Following Major Osteoporotic Fracture: Results from the Women’s Health Initiative Study

Michaela Juels BS, Joseph C. Larson MS., Kristine E. Ensrud, MD, MPH, Marcia L. Stefanick Ph.D., Aladdin H. Shadyab Ph.D., Lorena Garcia MPH, DrPH, Rami Nassir Ph.D., Peter F. Schnatz DO, FACOG, FACP, NCMP, Rebecca Nelson Ph.D., Carolyn J. Crandall, MD, MS, MACP

Author Affiliations:

Michaela Juels, BS: David Geffen School of Medicine, University of California, Los Angeles, USA.

Joseph C. Larson, MS: Fred Hutchinson Cancer Research Center, Seattle, WA, USA

Email: jlarson@whi.org

Kristine E. Ensrud, MD, MPH: Division of Epidemiology and Community Health and Department of Medicine, University of Minnesota, Minneapolis MN, USA.

Email: ensru001@umn.edu

Marcia L. Stefanick, PhD: Departments of Medicine (Stanford Prevention Research Center) and of Obstetrics & Gynecology, Stanford University, Stanford CA, USA.

Email: stefanick@stanford.edu

Aladdin H. Shadyab Ph.D: Herbert Wertheim School of Public Health and Human Longevity Science and Division of Geriatrics, Gerontology, and Palliative Care, Department of Medicine, University of California San Diego, La Jolla, CA. USA.

Email: ahshadya@health.ucsd.edu

Lorena Garcia, MPH, DrPH: Department of Public Health Sciences, School of Medicine, University of California, Davis, CA, USA.

Email: [lgarcia@ucdavis.edu](mailto:lgarcia@ucdavis.edu)

Rami Nassir Ph.D: Department of Pathology, School of Medicine, Umm Al-Qura University, Mecca, Saudi Arabia

Email: rmnassir@formerstudents.ucdavis.edu

Peter F. Schnatz, DO: Reading Hospital / Tower Health & Drexel University; Departments of Obstetrics, Gynecology and Internal Medicine, Philadelphia, PA. USA.

Email: peter.schnatz@towerhealth.org

Rebecca A. Nelson, PhD: City of Hope Comprehensive Cancer Center, Department of Computational and Quantitative Medicine, Division of Biostatistics, Duarte, CA. USA.

Email: rnelson@coh.org

Carolyn J. Crandall, MD, MS, MACP: Division of General Internal Medicine and Health Services Research, Dept. of Medicine, David Geffen School of Medicine at University of California, Los Angeles, USA. Email [ccrandall@mednet.ucla.edu](mailto:ccrandall@mednet.ucla.edu)

Corresponding Author: Michaela Juels

David Geffen School of Medicine at UCLA
Student Affairs Office
Geffen Hall, Suite 200 
885 Tiverton Drive, Box 951720
Los Angeles, CA, USA 90095-1720

[mjuels@mednet.ucla.edu](mailto:mjuels@mednet.ucla.edu)

Supplemental Figures

eFigure 1. 5-year Mortality KM by Race


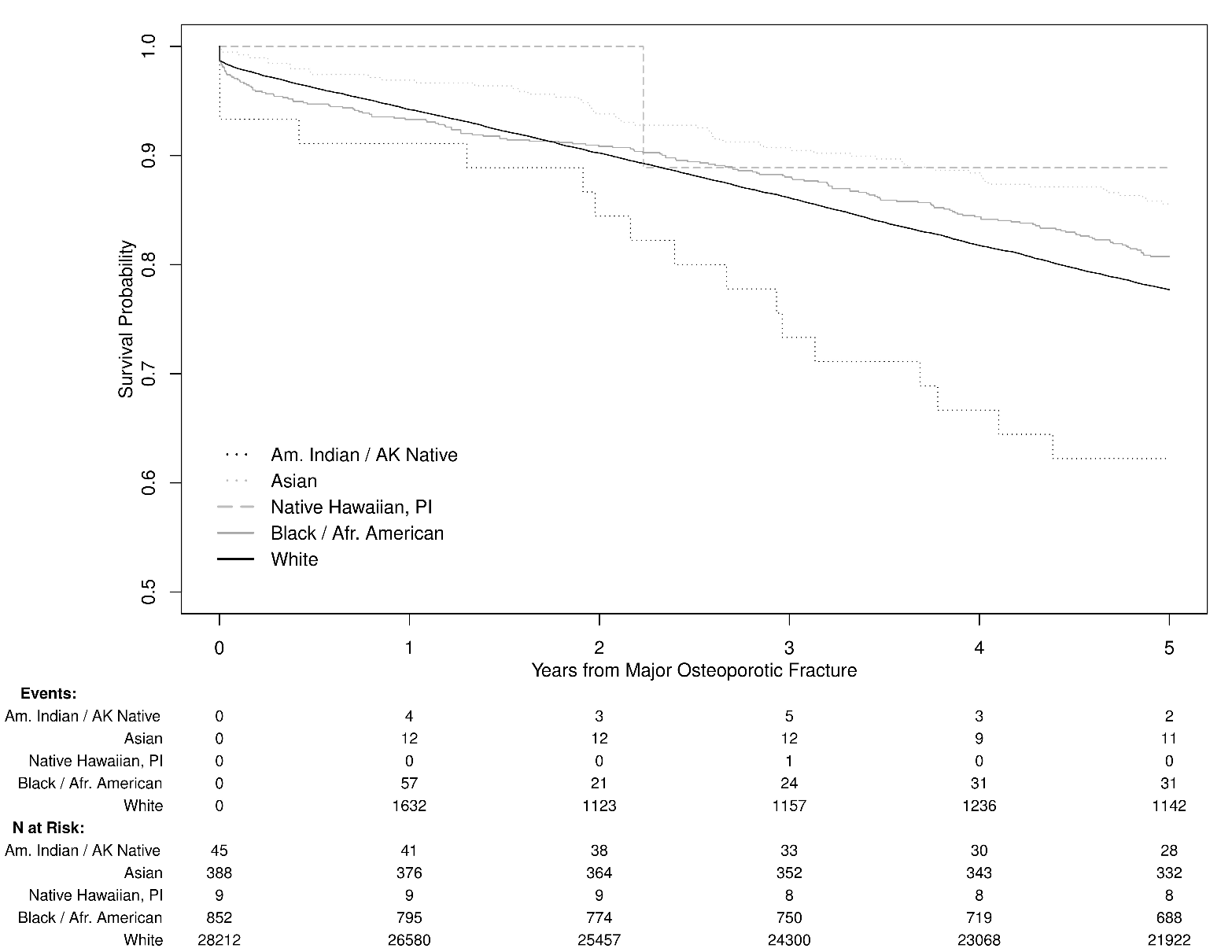


eFigure 2. 5-year Mortality KM by Ethnicity


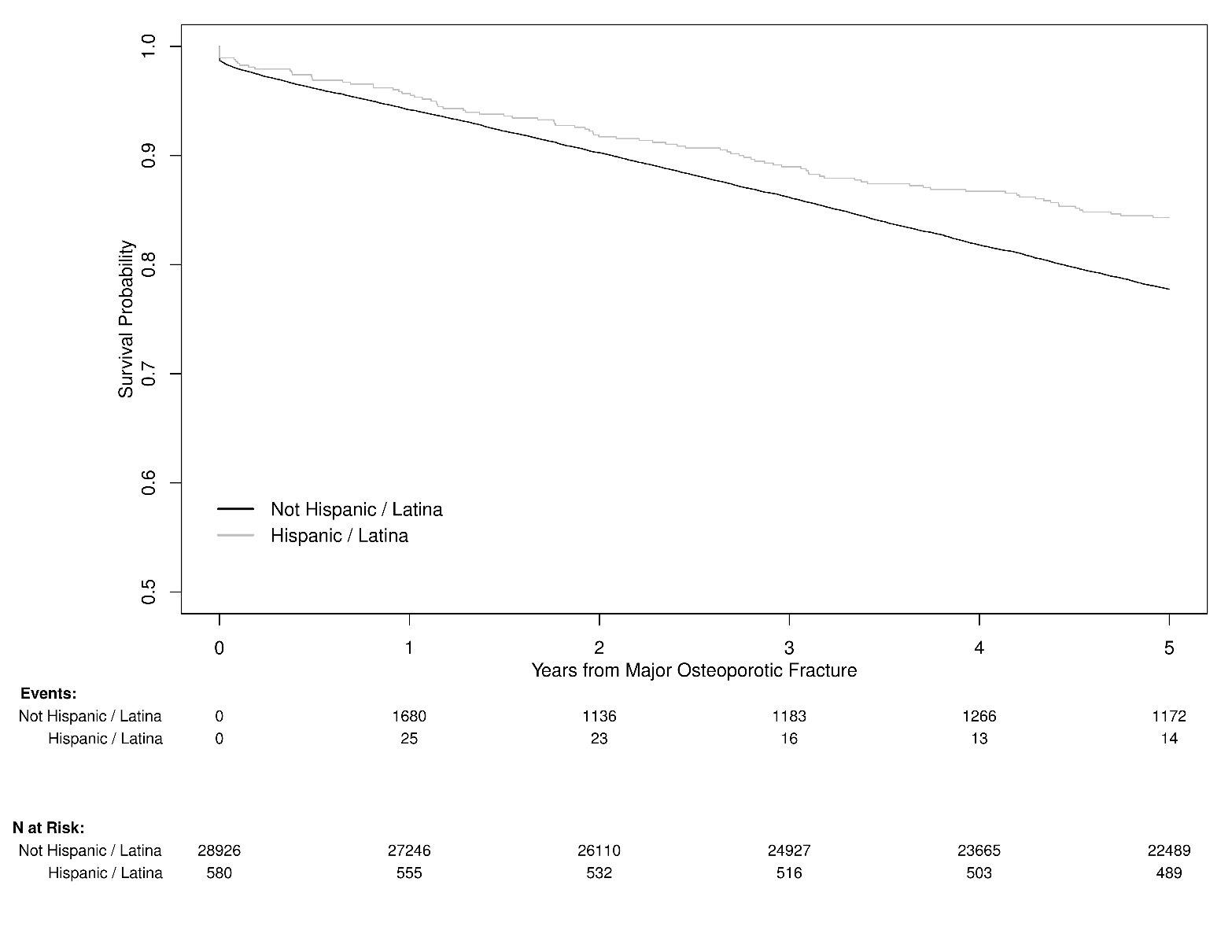

Supplement: Supplementary file 2 — Supplementary file2 (DOCX 424 KB) [file 11606_2025_9506_MOESM2_ESM.docx]
